# Bone Metastasis of Breast Cancer: Molecular Mechanisms and Therapeutic Strategies

**DOI:** 10.3390/cancers14235727

**Published:** 2022-11-22

**Authors:** Lulian Pang, Chen Gan, Jian Xu, Yingxue Jia, Jiaying Chai, Runze Huang, Anlong Li, Han Ge, Sheng Yu, Huaidong Cheng

**Affiliations:** 1 Department of Oncology, Anhui Medical University, Hefei 230601, China; 2 Center for Translational Medicine, Anhui Medical University, Hefei 230032, China; 3 Department of Oncology, Shenzhen Hospital of Southern Medical University, Shenzhen 518000, China

**Keywords:** breast cancer, bone metastases, molecular mechanisms, therapeutic strategies

## Abstract

**Simple Summary:**

In this review, we present knowledge of the molecular mechanisms and therapeutic strategies of bone metastasis of breast cancer, particularly the interaction between metastatic breast cancer cells and the bone microenvironment in promoting the development of bone metastasis in breast cancer patients, with the aim of improving the quality of life and prognosis of breast cancer patients and providing a reference for future research directions.

**Abstract:**

Bone metastasis is a common complication of many types of advanced cancer, including breast cancer. Bone metastasis may cause severe pain, fractures, and hypercalcemia, rendering clinical management challenging and substantially reducing the quality of life and overall survival (OS) time of breast cancer patients. Studies have revealed that bone metastasis is related to interactions between tumor cells and the bone microenvironment, and involves complex molecular biological mechanisms, including colonization, osteolytic destruction, and an immunosuppressive bone microenvironment. Agents inhibiting bone metastasis (such as bisphosphate and denosumab) alleviate bone destruction and improve the quality of life of breast cancer patients with bone metastasis. However, the prognosis of these patients remains poor, and the specific biological mechanism of bone metastasis is incompletely understood. Additional basic and clinical studies are urgently needed, to further explore the mechanism of bone metastasis and develop new therapeutic drugs. This review presents a summary of the molecular mechanisms and therapeutic strategies of bone metastasis of breast cancer, aiming to improve the quality of life and prognosis of breast cancer patients and provide a reference for future research directions.

## 1. Introduction

Breast cancer has surpassed lung cancer to become the most common tumor worldwide [1], and approximately 70% of patients with advanced breast cancer will develop bone metastasis [2]. The spine is the most common site of bone metastasis (BM) in patients with breast cancer [3]. Once bone metastasis occurs, it is rarely treated successfully and increases the risk of bone-related morbidities, such as pain, pathological fracture, hypercalcemia, and spinal cord compression, which substantially decrease the quality of life of cancer patients [4]. An understanding of the molecular mechanisms underlying bone metastases of breast cancer is the basis for developing effective targeted drugs and improving the quality of life of patients.

**Figure 1:**
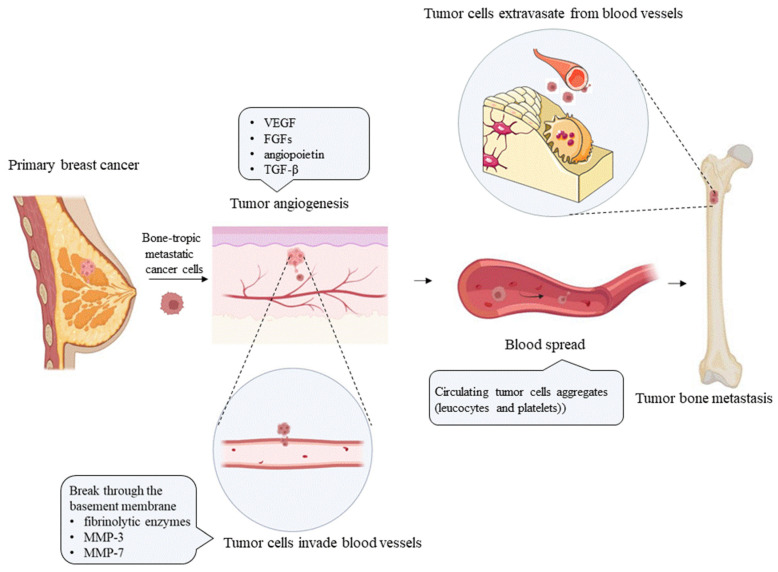
The process by which breast cancer cells migrate from the primary site to the bone. Tumor cells continue to proliferate and convert into a mesenchymal phenotype, and tumor angiogenesis is promoted upon stimulation with VEGF, FGFs, angiopoietin, and TGF-β [20]. Tumor cells interact with the surrounding stroma, to promote tumor cell migration, and intravasate through the vascular wall to enter the circulatory system, by producing fibrinolytic factors and MMP-3 and MMP-7. Circulating tumor cells interact with blood cells, such as lymphocytes, neutrophils, and platelets, to form multicellular aggregates and promote tumor survival. Metastatic cancer cells enter the bone via the blood circulation; arrest in distant capillaries; extravasate into the bone marrow through the vascular system; interact with cells in the bone microenvironment, such as osteoblasts and stromal cells; and then survive and activate osteoclasts; finally resulting in osteolytic metastasis. MMP-3 = matrix metalloproteinase 3; MMP-7 = matrix metalloproteinase 7; VEGF = vascular endothelial growth factor; FGFs = fibroblast growth factors; TGF-β = transforming growth factor beta.

Bone metastasis results from the mutual selection and interactions between metastatic tumor cells and the bone microenvironment, a concept consistent with the “seed and soil” hypothesis proposed by Paget in 1889 [5]. Tumor cells continue to grow, develop their own blood supply [6], and convert from epithelial cells to mesenchymal cells via the epithelial–mesenchymal transition (EMT), to acquire invasive activity that supports distant dissemination [7]. Tumor cells released from the primary tumor induce extracellular matrix (ECM) degradation through the production of fibrinolytic enzymes [8] and matrix metalloproteinases (MMPs) [9,10], and migrate to distant organs through lymphatic and blood vessels (as shown in Figure 1). However, successful progression to distant metastasis is very rare. In the blood circulation, cancer cells may interact with platelets [11] and leukocytes, to form aggregates that increase the resistance of cancer cells to shear stress in the bloodstream and protect them from immune cell-mediated clearance; moreover, most tumor cells do not pass through the pulmonary capillary bed. Venous blood from the pelvic region and breasts flows, not only into the vena cava, but also directly into the vertebral venous system [12,13]. By traveling through the vertebral venous system, cancer cells in the pelvic region and breasts may bypass the pulmonary circulation, which also explains the predisposition of breast and prostate cancer cells for developing axial bone metastasis. Moreover, bone marrow has a rich blood supply, and growth factors are released from the matrix during continuous remodeling [14]. These growth factors, such as transforming growth factor β (TGF-β) and insulin-like growth factors (IGFs) [15], attract metastatic cells. The surviving cancer cells cross the vessel wall and invade the bone marrow matrix, where they form their own blood supply. Tumor cells that colonize bone produce tumor-related factors, such as parathyroid hormone-related peptide (PTHrP), prostaglandins, and interleukins (ILs), which stimulate the differentiation and proliferation of osteocytes, to form bone metastases [16,17]. The final outcome of bone metastasis differs among tumor types. Bone metastasis in prostate cancer patients is mainly caused by the increased bone formation resulting from excessive osteoblast activity. In contrast, most patients with breast, lung, and kidney cancers mainly exhibit osteolytic bone metastases due to enhanced osteoclast activity [18,19]. When the bone matrix is dissolved, numerous bone-related factors, such as TGF-β, IGFs, fibroblast growth factors (FGFs), platelet-derived growth factor (PDGF), and bone morphogenetic proteins (BMPs), are released, to promote the growth and division of tumor cells, forming a vicious cycle of tumor-induced bone destruction [17].

In addition to activating osteoclasts to cause bone destruction, metastatic cancer cells regulate the immune system in the bone microenvironment. Myeloid-derived suppressor cells (MDSCs) accumulate through the influence of various tumor-derived factors, such as vascular endothelial growth factor (VEGF), TGF-β, a variety of ILs, and prostaglandin E2 (PGE2), which inhibit the tumoricidal effect induced by tumor recognition by immune effector T lymphocytes and promote the survival of tumor cells [20,21]. Therefore, exploring the interactions between tumor cells and the bone microenvironment is the key to understanding bone metastasis.

The main purpose of treatments for breast cancer bone metastasis, including radiotherapy (RT), chemotherapy, endocrine therapy, and surgery, is to prevent tumor progression and relieve symptoms. As research has continuously progressed, targeted drugs have been used to treat breast cancer bone metastasis and have provided significant benefits to patients. For example, denosumab, a human-derived monoclonal antibody (mAb) targeting receptor activator of nuclear factor kappa B ligand (RANKL), effectively inhibits bone transformation, reduces the risk of skeletal-related events (SREs), and is well tolerated [22]. This review summarizes the complex molecular interactions between tumor cells and the bone microenvironment during bone metastasis of breast cancer, to provide evidence and guidance for therapy.

## 2. Epidemiology and Detrimental Effects of Bone Metastasis on Patients with Breast Cancer

Bone metastases are commonly detected in patients with solid tumors of the prostate, lung, kidney, breast, or colon [23]. Bone is the most common site of metastasis in patients with metastatic breast cancer, and 60–75% of patients with metastatic breast cancer are first diagnosed with bone metastasis [18]. A population-based study showed that from 2010 to 2013, patients with an initial diagnosis of metastatic breast cancer involving the bone accounted for 3.6% of all patients with an initial diagnosis of breast cancer and 62.5% of patients with an initial diagnosis of metastatic breast cancer. Additionally, 70.5% of patients with bone metastases were hormone receptor-positive (HR+/human epidermal growth factor receptor 2 (HER2-): 57.6%; HR+/HER2+: 12.9%) [24]. In one systematic review and meta-analysis, 12% of patients with stage I–III breast cancer developed bone metastases during a 5 year follow-up, and a median of 55% of the patients who developed distant metastases during follow-up had bone metastases. Studies have used bone scans and sophisticated imaging techniques such as magnetic resonance imaging (MRI) or computed tomography (CT) to screen for the presence of bone metastases [25]. In one study, approximately 1.9% of patients had bone metastases in the first year after the initial diagnosis of breast cancer (95% CI: 1.7–2.0%), 20.3% of patients with advanced breast cancer had bone metastases in the first year after diagnosis, and 38.5% of breast cancer patients had SREs in the first year after the diagnosis of bone metastasis (95% CI: 36.0–41.0%) [26]. The cumulative incidence of SREs (defined as pathological fracture, spinal cord compression, surgery, or RT to bone) in patients with breast cancer was 47% in a 12 year Korean cohort study, which was higher than the 31.4% reported for patients with prostate cancer and 38.0% for patients with multiple myeloma [27]. Major SREs occur every 3–6 months, leading to a reduction in the quality of life of patients and eventually to death due to skeletal complications and their treatment [28]. The survival rate of breast cancer patients was better than that of patients with other solid tumors, particularly those with HR+ breast cancer. Patients with the HR+/HER-2- subtype had the highest survival rate (survival rate of 92.5% at 4 years), followed by those with the HR+/HER-2+ subtype (90.3%), HR-/HER2+ subtype (82.7%), while patients with triple-negative breast cancer had the lowest survival rate (77.0%, including early- and advanced-stage patients), which dropped to 11.2% among triple-negative breast cancer patients with stage IV [29]. Metastases are common in patients with triple-negative breast cancer, especially visceral metastases, and are associated with a poor prognosis [30]. The survival rate of patients with bone metastasis is relatively higher; the median survival time of breast cancer patients with bone metastasis depends on age, race, grade, breast cancer subtype, concurrent metastasis in other visceral sites, and treatment (surgery and chemotherapy), ranging from 13 to 47 months [31].

Bone pain was the most common complication of metastatic bone disease and was reported in 81.4% of patients with metastatic cancer [32]. The mechanism of metastatic bone pain is complex and includes increased osteoclast activity that leads to pathological changes in bone neuropathy and mechanosensitive pain caused by bone loss [33]. Periosteal infiltration by the tumor may also cause bone distending pain when stretching occurs [34]. In addition, pathological fracture, radiation, or surgery to bone, as well as spinal cord compression, may lead to bone pain [35]. The most common and fatal metabolic complication in cancer patients is hypercalcemia. Malignant tumor-related hypercalcemia is reported in 20–30% of cancer patients during the disease course; it is commonly observed in patients with breast cancer, multiple myeloma, squamous cell carcinoma, and other primary tumors, and predicts a poor prognosis [36,37]. The most common causes include malignant humoral hypercalcemia mediated by PTHrP, production of osteolytic cytokines, and excess 1, 25-dihydroxy vitamin D production [38]. Pathologic fracture is a relatively late complication of bone involvement associated with endocrine therapy drugs, such as aromatase inhibitors, and with bone metastasis; it occurs in 17–50% of women, resulting in pain, deformity, loss of mobility, paralysis, and death [39,40]. Tumor metastasis to bone increases the risk of cardiovascular events, among which venous thromboembolism (VTE), which is associated with increased mortality, has the highest incidence [41].

## 3. Risk Factors and Imaging Examinations for Bone Metastases in Patients with Breast Cancer

Bone metastasis in breast cancer patients has a characteristic age distribution; is related to clinicopathological features, the breast cancer subtype, gene expression, bone turnover markers (BTMs), and tumor markers; and can be detected using blood, urine, and imaging examinations, which provides guidance for its early detection and treatment. A retrospective analysis showed that middle-aged and elderly patients with breast cancer, mainly those aged 40–60 years old, were prone to bone metastasis [42]. Zhangheng Huang et al. conducted a retrospective analysis of patients with invasive ductal carcinoma presenting with bone metastasis, using a multivariate logistic regression model. The risk factors for bone metastasis of breast cancer included sex, primary site, grade, T stage, N stage, liver metastasis, brain metastasis, lung metastasis, and breast cancer subtype [43]. Koray Başdelioğlu found that the tumor diameter and positive axillary lymph nodes (ALNs) may play important roles in bone metastasis of breast cancer. The tumor diameter of the bone metastasis group was approximately 2–4 cm (3.07 ± 1.71 cm), and the incidence of bone metastasis was high in breast cancer patients with at least four axillary lymph node metastases [44]. A systematic review and meta-analysis by Huanmei Liu et al. showed that the risk of BM in patients with stage T4, T3, and T2 disease was 14.57 times, 4.74 times, and 1.99 times higher, respectively, than that in patients with stage T1 disease (OR = 14.57, OR = 4.74, and OR = 1.99, respectively; *p* < 0.5). The risk of bone metastasis in patients with lymph node metastasis was higher than that in patients without lymph node metastasis (OR = 2.60, *p* = 0.002), and the incidence of bone metastasis in patients without ductal or lobular disease was significantly higher than that in patients with ductal BC (OR = 1.26,95%, *p* = 0.001). However, the menopausal status and histological grade had no significant effect on the bone metastasis of breast cancer [45]. A retrospective study of 490 breast cancer patients with distant metastases suggested that luminal A tumors were more prone to bone-only metastases than HER-2 (+) and triple-negative breast cancers, which had a greater probability of brain metastases. Another retrospective study of 263 patients with primary invasive breast cancer accompanied by bone metastasis, who were stratified based on breast cancer subtypes, showed that ER+HER2- tumors with Ki67 score >13% were associated with the highest incidence of bone metastasis(87.8%) [46]. In a mouse model of bone metastasis, numerous genes controlling interferon regulatory factor 7 (Irf7) were suppressed in response to bone metastasis, which allowed tumor cells to escape immune surveillance and promoted the occurrence of bone metastasis [47]. In breast cancer cells, c-Src expression is essential for homing to the bone, essential for the survival and growth of these cells in the bone marrow, and associated with delayed bone metastasis occurring years after primary tumor resection [48]. Xu Teng et al. constructed a differentially expressed lncRNA-mRNA network associated with breast cancer bone metastases and identified core driver genes, including BNIP3 and the lncRNA RP11-317-J19.1, providing promising targets for the treatment of bone metastases [49].

BTMs reflect bone formation and resorption and have been used as risk factors for the occurrence of bone metastasis. Procollagen type I N-terminal propeptide (PINP) reflects the deposition of type I collagen and is used as a marker of bone formation in cancer patients. Some studies have used serum P1NP levels as a tool to evaluate the extent of bone involvement and detect bone metastases in breast cancer patients [50,51]. Windy Dee-Colomb et al. showed that a serum P1NP level greater than or equal to 75 ng/mL was associated with a shorter time to bone metastases and shorter overall survival (OS) of patients with stage I-III breast cancer [52]. Type 1 collagen is degraded by the proteases secreted by osteoclasts, which release N-terminal and C-terminal fragments that are detectable in both blood and urine (N-terminal cross-linking telopeptide, NTX, and C-terminal cross-linking telopeptide of type I collagen, CTX, respectively), and which are markers of bone resorption [53]. Elevated urinary α-CTX levels have been documented in breast cancer patients with bone metastases [54]. Berrin Papila Kundaktepe et al. tested serum NTX and urinary α-CTX levels in 30 postmenopausal patients with breast cancer presenting with bone metastases, 20 breast cancer patients without bone metastases, and 20 healthy postmenopausal women. Serum NTX and urine α-CTX levels in breast cancer patients were all higher than the standard values, and very high urine α-CTX levels were detected in breast cancer patients with bone metastases (specificity:70%; sensitivity: 85%), suggesting that urinary α-CTX level is a particularly important diagnostic marker of breast cancer [55]. Some limitations of BTM assessment still hinder its routine application. These factors include patient characteristics (age, sex, food intake, natural circadian variations, and liver and/or kidney disease) and concomitant treatments that interfere with bone turnover, such as hormone therapy, that are currently administered to breast cancer patients [56]. Phosphatase 5b (TRACP5b), secreted by osteoclasts during bone resorption, is considered a novel marker of bone resorption [57]. TRACP 5b has an additional advantage over other bone markers, because it does not show a strong dependence on food intake or circadian rhythms, and its activity is not affected by kidney or liver function [58]. Chuanbo Feng et al. showed that the AUC of TRACP5b in the diagnosis of breast cancer bone metastasis was 0.741 (sensitivity: 71.33%, specificity: 81.26%), that of cancer antigen (CA)-125 was 0.715 (sensitivity: 75.51%, specificity: 0.715, 45%), and the AUC of TRACP5b and CA125 combined was 0.758 (sensitivity 73.12%, specificity 88.17%). Therefore, TRACP5b and CA125 may be new biomarkers for the diagnosis of bone metastasis [59]. In a retrospective study by Akram Yazdani et al., carcinoembryonic antigen (CEA) and CA-153 levels were not significantly different in breast cancer patients with or without bone metastasis, and serum concentrations of alkaline phosphatase (ALP) (OR = 1.005) were an independent risk factor associated with bone metastases [60]. Bone alkaline phosphatase (BAP) and ALP were used to screen for bone metastases in patients with solid tumors, with a specificity of 69% and 100% and a sensitivity of 86% and 52%, respectively [61]. Bone morphogenetic protein 7 (BMP7) is overexpressed in primary breast cancer and has also been identified as being associated with an increased risk of bone metastasis [62].

The diagnosis of bone metastasis is based mainly on imaging techniques, including X-ray imaging, skeletal scintigraphy (SS), CT, MRI, and [18F]-fluorodeoxyglucose-positron emission tomography with integrated computed-tomography (FDG-PET/CT). X-ray imaging is a first-line tool to investigate painful areas and confirm a positive diagnosis of suspected pathological fractures in symptomatic cancer patients; however, its sensitivity is very low, especially for the detection of lytic lesions within bone trabeculae, in which 30–75% bone loss is required for detection, and therefore it has a limited role in systematic screening of bone lesions [63]. SS relies on the use of a tracer (diphosphonate-bound technetium-99 m) with affinity for osteoblasts, which limits the sensitivity of SS for the diagnosis of osteolytic bone disease. CT is more sensitive than X-ray imaging for the diagnosis of bone metastasis, but the target site is limited to specific anatomical locations and this method involves radiation and is thus generally not used for systematic bone screening. For detection using a CT examination, bone metastases must be at least 1 cm in size, with a bone density loss of approximately 25 to 50%. MRI is independent of osteocyte activity and is sensitive to early changes in bone marrow [64]. The characteristic MRI pattern of bone metastases is typically a low T1 signal, high T2 signal, and signal restriction on diffusion-weighted imaging (DWI) [65]. MRI enables the visualization of lesions with high accuracy, and it also facilitates the evaluation of spinal cord integrity and ultimately the compression status. The radiation-free and whole-body assessment (i.e., WBMRI) capabilities of MRI have resulted in the promotion of MRI as an important tool for many clinicians involved in the detection of bone neoplastic lesions [63]. PET/CT is currently considered a good alternative method for the detection of distant metastases, especially bone metastases, because it is superior to SS in detecting osteolytic lesions, and its detection accuracy for bone metastases is higher than that of CT; therefore, it is often used to screen for breast cancer metastasis [66,67].

## 4. Effect of Estrogen Receptor (ER) Positivity on Promoting Bone Metastasis

More than 70% of breast cancers are HR+ [24]. Clinical evidence shows that ER+ breast cancer subtypes have a higher propensity for bone metastasis [68], while HR- breast cancer subtypes tend to spread to visceral organs, such as the lungs and liver [69]. Cletus A. et al. reported that ER+/HER2+ and ER-/HER2+ breast cancers had different metastasis patterns, and that the OS of patients with the ER+/HER2+ signature was better than that of patients with the ER-/HER2+ signature, particularly those treated with anti-HER-2 therapy [70]. Therefore, exploring the intrinsic factors of ER subtypes is beneficial to better understand the mechanisms of bone metastasis of breast cancer and provide a greater survival benefit to patients [71]. ERα and ERβ are expressed at high levels in osteoblasts and osteoclasts, and are involved in bone metabolism [72,73]. Although ERα and ERβ are structurally similar, they differ in their functions. ER knockout (ERKO) model mice show bone alterations, accompanied by genital changes. ERα KO mice exhibit altered reproductive organ development and shorter longitudinal bone growth than ERβ KO mice [72], while ERβ KO mice exhibit longer bone growth and altered trabecular formation compared with ERα KO mice [73,74]. However, bone mass does not differ significantly between double ERα/β knockout mice, suggesting a mutual antagonism between ER isoforms.

The identification of liquid biopsy biomarkers in breast cancer patients with a high risk of bone metastasis showed that the exosomal miR-19a and integrin binding salivary protein (IBSP) secreted by bone-tropic ER+ breast cancer cells were significantly upregulated. IBSP attracts osteoclasts and forms an environment rich in osteoclasts in bone, facilitating the delivery of exosomal miR-19a to osteoclasts and thereby inducing osteoclastogenesis and creating a microenvironment conducive to the colonization of cancer cells [75]. In a mouse model of estradiol (E2)-dependent bone metastasis of ER+ breast cancer, ERα-induced tumor secretion of the osteolytic factor PTHrP, the number of osteoclasts at the bone–tumor interface, and osteolytic bone destruction were increased in an E2-dependent manner, which may explain the tendency of ER+ tumors to form osteolytic lesions [76]. The use of antiestrogen drugs, such as aromatase inhibitors and tamoxifen, has significantly improved the survival rate of patients [77,78]. However, the detailed mechanism by which ER promotes breast cancer bone metastasis remains unclear.

## 5. Bone Colonization of Breast Cancer Cells

### 5.1. Genetic/Epigenetic Changes

During the early stage of distant metastasis of primary tumors, tumor cells undergo genetic and epigenetic changes that promote their invasion ability, entry into the circulatory system from the primary tumor site, and ultimate colonization of sites conducive to survival and growth [79]. Steven L. Wood identified a series of loci that may be associated with the risk of bone metastasis through genome-wide association studies. One identified SNP (RS10771399) is located in the chromosome 12p11 region, which contains the PTHrP gene and is an important mediator of the vicious cycle of bone destruction. Therefore, the presence of this SNP may explain some of the known genetic heritability to promoting the development of bone metastases in patients with breast cancer [80]. Yibin Kang et al. analyzed and functionally verified genes overexpressed in the human breast cancer cell line MDA-MB-231 with high metastatic activity. These genes, including IL-11, connective-tissue-derived growth factor, MMP1, and chemokine-receptor CXCR4, synergistically lead to osteolytic metastasis, and most of them encode secreted and cell surface proteins. Two of these genes, IL-11 and CTGF, encode bone osteolytic and angiogenic factors, and their expression is further increased by the premetastatic cytokine TGF [81]. In addition, silencing or reducing the expression of certain genes promotes bone metastasis of spreading tumor cells. A study reported that decreased CTNND1 expression is associated with bone metastasis of triple-negative (TN) breast cancer and is involved in EMT and bone homing, by upregulating CXCR4 through the activation of the PI3K/AKT/HIF-1α pathway [82]. DNA methylation is an epigenetic modification that is detected using advanced DNA sequencing techniques, such as bisulfite sequencing, enabling analysis of the patterns of cytosine methylation in CpG islands present in gene promoters [83]. Differentiation related gene-1 (DRG-1) has been shown to inhibit the metastasis of various tumors [84,85]. Sucharita Bandyopadhyay observed that DRG-1 expression was reduced in breast cancer cells, especially in cells from patients with bone metastases. In the process of exploring the mechanism of transcriptional inactivation, the authors found that the DRG-1 gene has two potential CpG islands upstream of the transcription start site, and treatment with the demethylating agent 5-azacytidine increases DRG-1 expression, which revealed the role of methylation in promoting breast cancer bone metastasis [86]. Therefore, identifying genes with a high risk of bone metastasis in breast cancer patients has an important guiding role in discovering and targeting bone metastasis.

### 5.2. The Premetastatic Niche

The concept of a premetastatic niche is based on the idea that distant metastatic sites provide favorable conditions for the metastatic colonization of primary tumor cells. Primary tumor cells modulate bone marrow, to facilitate tumor cell localization and colonization, by producing circulating factors that regulate cells in the bone microenvironment; for example, heparanase and PTHrP expressed by breast cancer cells promote bone resorption in the absence of detectable bone metastasis [87,88]. OPN derived from the tumor cell matrix regulates tumor-associated macrophages (TAMs), to promote tumor angiogenesis and form an immunosuppressive microenvironment in osteoma [89,90]. Regulatory T (Treg) and Th17 cells (two subsets of CD4+ cells) in the tumor microenvironment regulate the production of cytokines and chemokines, promote the recruitment and functional activation of immune cell populations, and thus accelerate tumor progression and metastasis [91]. Claudia Tulotta and other researchers discovered that tumor-derived IL-1β limited primary tumor development, reestablished the infiltration of immune cells with potential antitumor functions, and supported breast cancer metastasis, by enhancing the motility and inhibiting the proliferation of tumor cells [92]. Furthermore, IL-1β stimulates γδT cells to express IL-17, resulting in the systemic expansion and polarization of neutrophils in mice with mammary tumors and endowing them with the ability to inhibit cytotoxic CD8+ T lymphocytes, thereby promoting tumor metastasis [93]. According to recent studies, the circulating exosomes in breast cancer patients with bone metastasis are rich in core binding factor subunit β (CBFB), which regulates bone metastasis markers, changes endogenous oxidative stress levels, and promotes bone metastasis in breast cancer patients [94,95].

Hematopoietic stem cells (HSCs) differentiate into myeloid progenitor cells and immature myeloid cells through the actions of cytokines and growth factors [96]. A small number of immature myeloid cells are transformed into MDSCs under the pathological conditions generated by the tumor microenvironment. These cells differentiate into TAMs, tumor-associated dendritic cells (TADCs), and tumor-associated neutrophils (TANs), which protect tumor cells from immune surveillance by inhibiting natural killer (NK) cells and CD8+ T cells [17]. Regarding the mechanism of MDSC recruitment and activation, in addition to activation of the JAK-STAT signaling pathway by GM-CSF, IL-6, and VEGF, Dickkopf-1 (Dkk1) promotes the accumulation of MDSCs and downregulates β-catenin expression in MDSCs from mice and humans, all of which exert an immunosuppressive effect on tumor progression [97,98]. In addition, MDSCs release VEGF, basic FGF (bFGF), the VEGF analog Bv8, and MMP-9, to ensure that the microenvironment is conducive to tumor cell growth [99]. After premetastatic niche formation, MDSCs interact with tumor cells through the IL-6 receptor (IL-6R) and epidermal growth factor receptor (EGFR), thereby inducing the progression of bone metastasis of cancer cells [100].

### 5.3. Tumor Cell Homing to Bone

Breast cancer cells escape from CD8+ T cell and NK cell-mediated immune responses and then intravasate into the circulatory system through the peripheral stroma, and tumor cells arrested in microvessels at metastatic sites escape from these vessels. The VEGF expression in tumor cells and osteoblasts is increased, thereby promoting bone neovascularization and the colonization of metastatic breast cancer cells in the bone microenvironment [101]. The detection of circulating tumor cells (CTCs) in the bloodstream and disseminated tumor cells (DTCs) in the bone marrow provide evidence of dissemination and colonization resulting in bone metastasis [102]. The theory that disseminating tumor cells colonize the bone marrow niche is similar to that of HSC homing. During homing, the CXCR4 expressed by HSCs and breast cancer cells binds to the CXC chemokine ligand 12 (CXCL12) expressed by bone marrow stromal cells, such as osteoblasts, endothelial cells, and fibroblasts, to retain HSCs or tumor cells in the bone marrow [103]. CXCL12-abundant reticular (CAR) cells are encapsulated around or located in the vicinity of sinusoidal endothelial cells and appear to be a key component of the osseous niche that provides a protective barrier to tumor cells [104]. During bone colonization, bone marrow-derived IL-1β induces NF-κB and CREB signaling in breast cancer cells, leading to autocrine Wnt signaling and CSC colony formation, thereby stimulating breast cancer cell colonization of bone, while inhibiting this pathway might prevent bone metastasis of breast cancer cells in vivo [105].

## 6. Bone Resorption Induced by Metastatic Breast Cancer Cells

### 6.1. Signal Transduction between Tumor Cells and Osteocytes

#### 6.1.1. PTHrP Produced by Tumor Cells

The mechanism of breast cancer cell-induced bone lysis has been gradually explored. Bone destruction is mainly caused by osteoclast activation mediated by cytokines and induced by tumor cells rather than by the direct effects of tumor cells on osteoclasts. Previous studies have shown that IL-1, IL-6, IL-8, PTHrP, tumor necrosis factor-alpha (TNF-α), PGE2, and macrophage inflammatory protein-1-alpha (MIP-1) cause bone destruction in patients with tumors [18,106]. PTHrP appears to be produced by breast cancer cells and mainly upregulates RANKL expression in osteoblasts or stromal cells, leading to osteoclast formation. A higher expression of PTHrP was detected in breast cancer cells in the bone microenvironment than in breast and soft tissues [107]. However, local nuclear PTHrP depletion in primary breast cancer is associated with adverse clinical outcomes through the prolactin–STAT5-PTHRP axis. This view contrasts with the previous theory that the PTHrP protein is often overexpressed in malignant breast tumors [108]. Therefore, the prognostic value of PTHrP protein overexpression in primary breast cancer remains controversial. Archana Kamalakar et al. reported that PTHrP (12–48), the first identified circulating fragment of PTHrP, is expressed by primary and metastatic breast cancer cells and has specific and potent biological activities. As a circulating biomarker, PTHrP (12–48) is significantly associated with breast cancer bone metastasis and locally regulates the differentiation of hematopoietic cells and the activity of osteoclasts in the tumor–bone marrow microenvironment, to promote tumor bone metastasis [109].

#### 6.1.2. RANKL–RANK Axis

**Figure 2:**
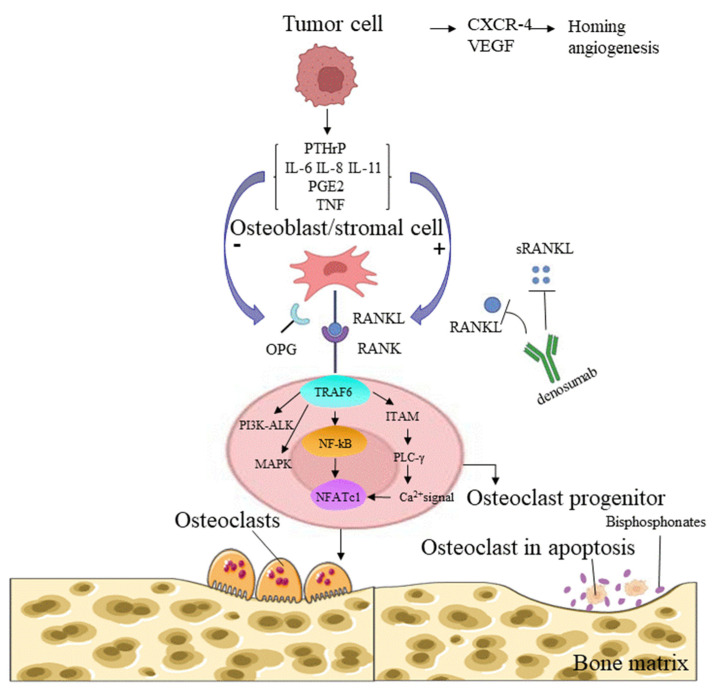
RANKL-RANK signaling in osteolytic bone metastases. Tumor cells arriving in the bone marrow microenvironment secrete tumor-related factors, such as CXCR-4 and VEGF, to promote the colonization and survival of tumor cells in the bone marrow, and PTHrP, IL-6, IL-8, IL-11, PGE2, and TNF stimulate osteoblasts and stromal cells to produce RANKL, which binds to the RANK expressed on osteoclasts. The interaction of RANKL and RANK promotes osteoclast survival and differentiation, and mature osteoclasts eventually cause bone resorption. Osteoblasts also produce OPG, which inhibits bone resorption by competing with RANK to bind to RANKL. The binding of the human monoclonal antibody denosumab to RANKL inhibits bone destruction, by blocking the RANKL-RANK axis. Bisphosphonates inhibit osteoclast activity and the growth of tumor cells, and exert an osteoprotective effect [121]. CXCR-4 = chemokine (C-X-C motif) receptor 4; VEGF = vascular endothelial growth factor; PTHrP = parathyroid hormonerelated peptide; IL-6 = interleukin-6; IL-8 = interleukin-8; IL-11 = interleukin-11; PGE2 = prostaglandin E2; TNF = tumor necrosis factor; RANKL = receptor activator of NF-kappa B ligand; RANK = receptor activator of NF-kappa B; OPG = osteoprotegerin.

Osteoclasts are formed from RANKL-stimulated bone marrow monocyte/macrophage lineage cells in the presence of macrophage colony-stimulating factor (M-CSF) [110]. M-CSF induces the proliferation of osteoclast precursor cells, supports their survival, and upregulates RANK expression, which is a prerequisite for the formation of osteoclast precursor cells [111,112]. RANKL and RANK are a receptor-ligand pair in the TNF receptor (TNFR) superfamily that regulates osteoclast development and bone metabolism [113] (Figure 2). According to recent studies, bone resorption factors, such as vitamin D3, IL-1, IL-11, IL-17, PTHrP, and TNF-α, act on osteoblasts to induce the RANKL expression that binds to RANK on the surface of osteoclasts and initiates signal transduction. RANK then recruits the adaptor TNF receptor-associated factor 6 (TRAF6), leading to the activation of NF-κB [114]. RANKL-induced NF-κB and c-Fos activation is important for the initial induction of nuclear factor of activated T cells cytoplasmic 1 (NFATc1), a key transcription factor for osteoclast formation activated by calcium signaling [114,115]. Calcium signaling is triggered by the phosphorylation of adaptors containing an immunoreceptor tyrosine-based activation motif (ITAM), and this event leads to the recruitment of splenic tyrosine kinase (Syk), phospholipase C gamma (PLCγ) activation, and subsequent calcium mobilization [116,117] (Figure 2). An in vitro study using cultured osteoclasts identified transmembrane protein 64 (Tmem64) as a positive regulator of Ca^2+^ oscillations during osteoclast production, through its interaction with sarcoplasmic endoplasmic reticulum Ca2+ ATPase 2 (SERCA2) [118]. The important contribution of RANKL/RANK to the bone microenvironment and immune system during bone metastasis reinforces the hypothesis that the loss of RANK/RANKL signaling is a critical therapeutic target in cancer. With the discovery of specific RANK signals in osteoclasts, human IgG2 monoclonal antibodies, such as denosumab, have been used to inhibit osteoclast activity, prevent bone metastasis of solid tumors or SREs, and prolong the survival of patients with tumors, and they may, to a certain extent, prevent the incidence of breast cancer in patients at high risk [119,120].

The RANKL–RANK axis is associated with the immune system. RANKL was originally identified in the mouse thymoma cell line EL40.5. A genomic analysis identified RANKL as a DC stimulator expressed by activated T cells [122], and RANKL was reported to be involved in regulating the interaction between T cells and DCs [123]. The selective activation and suppression of immunity by the RANKL–RANK interaction depend on the tissue location and pathophysiological background. In the bone microenvironment, tumor-stimulated RANKL overexpression contributes to the development of RANK+ osteoclasts and immunosuppression [124]. Studies have shown that human osteoclasts cultured in vitro induce immunosuppression, by suppressing the production of T cell-derived interferon gamma (IFN-γ) and CD40 ligands [125]. Although no long-term clinical studies have prospectively evaluated the efficacy of denosumab combined with immune checkpoint inhibitors (ICIs) targeting CTLA4, programmed cell death protein 1 (PD-1), and programmed cell death 1 ligand 1 (PD-L1) in the treatment of tumor bone metastases, some retrospective analyses and case reports have documented the synergistic effect of ICIs combined with denosumab. In mouse models, a combination therapy targeting CTLA4 and RANKL inhibited metastasis more effectively than either drug alone [119]. Inhibition of RANKL has been shown to enhance the efficacy of anti-PD-1/PD-L1 monoclonal antibodies in mouse models of prostate cancer, colorectal cancer, and melanoma [126]. In one case report, a 39-year-old patient with malignant melanoma accompanied by bone metastases responded significantly to denosumab plus the anti-CTLA-4 antibody ipilimumab [127]. In another case series, 10 patients with metastatic melanoma (including bone metastases) treated with denosumab and ICIs (mainly an anti-PD-1 antibody with or without an anti-CTLA-4 antibody) had an objective response rate (ORR) of 60% and a disease control rate (DCR) of 80%, with a median duration of 9.8 months of concurrent treatment [123]. The mechanism underlying the synergistic effects of anti-RANKL antibodies and ICIs remains unclear and appears to depend on the presence of activated Fc receptors and lymphocytes, particularly NK cells and CD8+ T cells [128]. Therefore, the combination of denosumab and ICIs is promising for improving antitumor efficacy and treating bone metastases, although the molecular mechanism of the interaction requires further clarification.

Molecular interactions that interfere with the RANKL–RANK signaling pathway are still being investigated. Jian Luo et al. found that leucine-rich repeat G-protein-coupled receptor 4 (LGR4; also called GPR48), another receptor for RANKL, competes with RANK for RANKL binding and inhibits classical RANK signaling during osteoclast differentiation, by activating the Gα_q_ and GSK3-β pathways [129]. Mikihito Hayashi et al. found that the binding of semaphorin 3A (Sema3A), an axon guidance molecule produced by osteoblasts, to neuropilin-1 (Nrp1) inhibited RANKL-induced osteoclast differentiation in a mouse model, by inhibiting ITAM and RhoA signaling. In addition, the interaction of Sema3A and Nrp1 stimulates osteoblast differentiation through the classical Wnt/b-catenin signaling pathway. The osteoprotective factor Sema3A provides the molecular basis for the development of compounds with anti-osteoclastic and pro-osteogenic properties [130]. In addition, new reports show that the osteoclasts stimulated by RANKL divide into daughter cells called osteoid cells after the completion of resorption and apoptosis, and participate in regulating bone resorption; moreover, inhibition of RANKL blocks this cell division process [131].

### 6.2. Osteolytic Destruction Induced by Osteoclasts

Osteoclasts break through the quiescent bone surface covered by the inner membrane and lining cells, and form an absorbing seal on the mineralized bone matrix [132], which, in combination with collagen dissolution, leads to bone matrix demineralization and degradation [133]. Many studies have reported that MMPs and cathepsin K, a very effective collagen-soluble cysteine protease, are the rate-limiting proteases for collagen matrix degradation [133,134]. Further studies showed that osteoclast-derived MMP-7 significantly contributed to tumor growth and tumor-induced osteolysis [135]. Through the action of osteoclasts, the bone matrix releases numerous growth factors and Ca^2+^, which promote the growth and invasion of tumor cells and then forms a vicious cycle of bone destruction [18,136].

## 7. Bone Microenvironment

**Figure 3:**
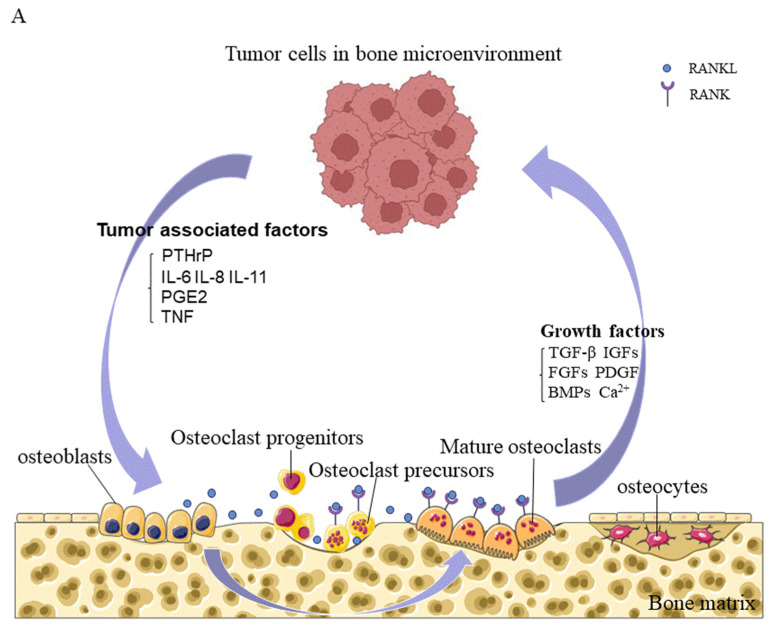

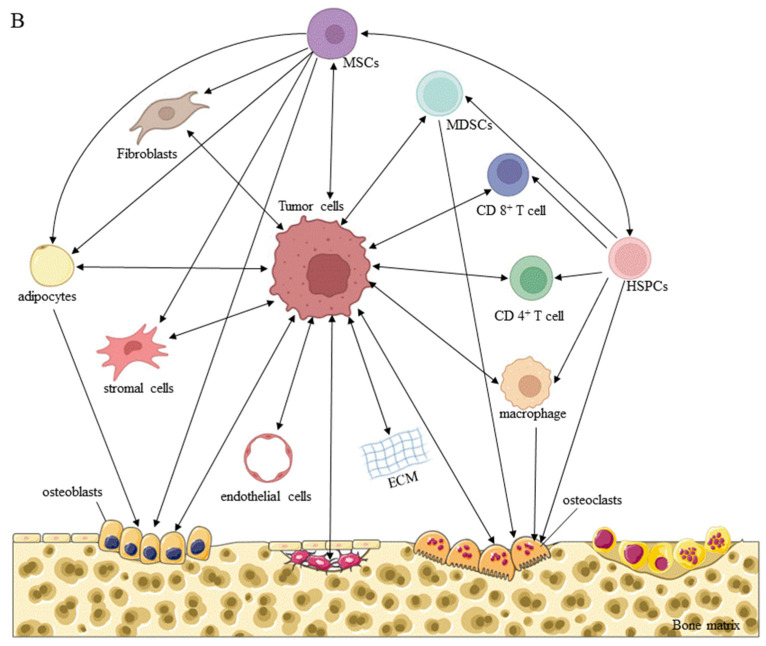
Interactions of tumor cells with the bone microenvironment. (**A**). Tumor cells in the bone microenvironment secrete tumor-related factors to act on osteoblasts, and the interaction of RANKL produced by osteoblasts with RANK expressed on osteoclasts stimulates osteoclast maturation and differentiation, leading to metastatic bone destruction. Bone resorption results in the release of various growth factors, such as TGF-β, IGFs, FGFs, PDGF, and BMPs, as well as Ca^2+^, which further promote the growth of tumor cells and form a vicious cycle of tumor growth–bone destruction. (**B**). A broader conception of this vicious cycle is the interactions between tumor cells and multiple cells in the bone microenvironment, which jointly participate in osteolytic bone destruction: mesenchymal skeletal stem cells (MSCs) produce osteoblasts, stromal cells, fibroblasts, and adipocytes; MSCs also promote the expansion and homing of hematopoietic stem cells (HSCs). Hematopoietic stem and progenitor cells (HSPCs) produce CD8+ T cells, CD4+ T cells, MDSCs, macrophages, and osteoclasts. CD8+ T cells directly kill recognized tumor cells and inhibit tumor growth. CD4+ T cells inhibit immune functions. MDSCs exert an immunosuppressive effect through the actions of a variety of tumor derived factors, such as VEGF, TGF-β, and PGE2, and differentiate into osteoclasts. Macrophages interact with tumor cells and differentiate into osteoclasts. VEGF is produced by tumor cells or endothelial cells upon tumor stimulation, to form a new blood supply that promotes tumor growth and reproduction. The ECM contains various growth factors, such as TGF-β and IGFs, which are released as a result of osteoclast resorption, to regulate the growth of tumor cells. PTHrP = parathyroid hormone-related peptide; IL-6 = interleukin-6; IL-8 = interleukin-8; IL-11 = interleukin-11; PGE2 = prostaglandin E2; TNF = tumor necrosis factor; TGF-β = transforming growth factor beta; IGFs = insulin-like growth factors; FGFs = fibroblast growth factors; PDGF = platelet-derived growth factor; BMPs = bone morphogenetic proteins; VEGF = vascular endothelial growth factor; ECM = extracellular matrix.

The bone microenvironment is a unique, complex compartment that houses host bone cells (osteoblasts, osteocytes, osteoclasts, and their precursors), stromal cells, hematopoietic and immune cells, fibroblasts and endothelial cells, adipocytes, and ECM containing numerous growth factors or signaling factors. The classic vicious cycle of bone metastasis is formed by interactions between tumor cells and osteoclasts. Briefly, osteoclasts are activated by tumor cells, to promote bone resorption through direct or paracrine activation, and the growth factors released from the bone matrix promote the growth of tumor cells, thereby aggravating bone destruction [137] (Figure 3).

### 7.1. Immune Regulation of Immune Cells in the Bone Microenvironment

In addition to the interactions between tumor cells and osteoclasts, immune cells, particularly T lymphocytes, play an important role in the osteolytic bone destruction caused by bone metastasis of breast cancer cells [138]. The premetastatic niche of breast cancer bone metastasis is mimicked in mice bearing invasive breast tumors. DCs presenting primary tumor antigens activate CD4+ CD8+ T cells in the lymph node drainage area, stimulating the expression of RANKL and IL-17F, as well as migration to the bone marrow. Simultaneously, the expression of IL-23 stimulates the activation of CD4+ T cells and Th17 cells in the bone microenvironment and activates osteoclasts, to cause bone destruction. In contrast, CD8+ T cells derived from the mouse bone marrow microenvironment express the antiosteoclastic cytokines IFN-γ and IL-10 at high levels, and CD8+ FoxP3+ Treg cells derived from bone marrow inhibited the tumor-specific CD4+ T-cell phenotype before the arrival of tumor cells in bone marrow in an orthotopic mouse model, exerting a protective effect during the maintenance of bone homeostasis [139]. Immune cells not only act directly or indirectly on osteoclasts, but also regulate the immunosuppressive bone microenvironment, to promote tumor survival and growth. Recent studies have shown that expanded PD-L1+ monocytic myeloid-derived suppressor cells (M-MDSCs) in the microenvironment effectively inhibit T cell activation, possibly through their production of immunosuppressive factors such as NO and their expression of PD-L1, as the receptor of PD-1 is present in more than 70% of T cells in bone [140]. In addition, MDSCs, also called "natural suppressor cells", accumulate under the influence of diverse tumor-derived factors, such as VEGF, TGF-β1, ILs, and PGE2; MDSCs not only inhibit a variety of immune effectors, especially CD4+ and CD8+ T lymphocytes, but also transform into osteoclasts, which promote tumor progression and tumor-induced bone loss [21,141].

### 7.2. Promotion of a Vicious Cycle of Metastatic Bone Destruction by Growth Factors

Osteoclast-mediated bone destruction results in the release of growth factors and calcium from the bone matrix into the bone microenvironment. Ninety percent of the protein released is collagen, and the remaining ten percent is composed of growth factors, including TGF-β, IGFs, FGFs, PDGF, and BMPs. These growth factors stimulate the growth of tumor cells and alter their phenotype, provide a favorable microenvironment for metastatic cell invasion by disrupting the bone metabolic balance and immunity, and promote a vicious cycle of tumor metastasis and bone destruction.

TGF-β is one of the most abundant growth factors in the bone matrix and is released locally in the microenvironment in an active form during tumor-induced osteoclastic bone resorption. TGF-β increases the expression of PTHrP in tumor cells via the TGF-β-Smad signaling pathway and enhances osteoclast generation and bone destruction by upregulating the expression of RANKL in adjacent osteoblasts, which is crucial for establishing the vicious cycle of tumor cell metastasis and bone destruction [142,143,144]. In addition, TGF-β promotes the EMT; enhances cell invasiveness, angiogenesis, and metastatic progression; and suppresses immune responses. Blocking TGF-β signaling disrupts the vicious cycle, reverses the EMT and enhances immune responses [145]. X Meng et al. found that bone marrow-specific TGF-β signaling mediates bFGF expression in bone through the downstream mitogen-activated protein kinase (MAPK)-extracellular signal-regulated kinase (ERK)-cFos pathway, to promote the bone metastasis of breast cancer [146]. Integrin β3 (iβ3) alters gene expression and bone destruction by regulating Gli2 and PTHrP in a TGF-β-dependent manner [147]. In addition, enhancer of zeste homolog 2 (EZH2) coactivates TGF-β signaling through the integrin β1 (focal adhesion kinase (FAK) pathway, to promote the bone metastasis of breast cancer [148]. Therefore, TGF-β blockers are key agents that interrupt the vicious cycle of bone destruction [149].

IGFs are important regulators of bone remodeling and metabolism, and play important roles in cancer metastasis [150]. IGFs promote the homing, dormancy, colonization, and spread of bone metastatic tumors, by binding to the IGF-I receptor (IGF-IR). In the mouse model established with the bone-seeking clone (MDA-231BO) of the human breast cancer cell line MDA-MB-231, IGF-I significantly promoted the anchorage-independent growth of MDA-231BO tumors. In addition, compared with parental MDA-231 (MDA-231P) cells, MDA-231BO cells produced more PTHrP, either in the absence or presence of TGF-β, which increased their survival and growth in bone, and promoted bone resorption [151]. Moreover, in an animal model, bone-derived IGF-I and IGF-IR physically interacted to activate the serine/threonine kinase Akt and transcription factor NF-κB and subsequently stimulated bone metastasis of MDA-MB-231 human breast cancer cells, by inducing their proliferation and inhibiting their apoptosis [15].

### 7.3. Inhibition of Lytic Bone Destruction by Osteoprotegerin (OPG)

OPG is a soluble "bait receptor" for RANKL that plays an important role in bone biology and the immune system. The increased bone mineral density in transgenic mice expressing OPG may be due to the decreased number of osteoclasts resulting from a reduction in their production, providing evidence that OPG inhibits osteoclast differentiation and reduces bone resorption [152]. Upregulation of RANKL and OPG aggravates osteoclast-induced bone destruction. However, the tumor-inducing signals that cause the imbalance are diverse, and this diversity may be related to the source of RANKL and OPG production and the heterogeneity of metastatic tumor cells [19,153]. The IL-1, IL-6, IL-8, PTHrP, TNF-α, and MIP-1 secreted by metastatic breast cancer cells stimulate osteoblasts and stromal cells to secrete RANKL and subsequently affect osteoclast production in the bone microenvironment [154,155]. However, factors secreted by tumor cells, such as PTHrP, IL-1, and PGE2, have been shown to act on the bone matrix and stimulate osteoclast activity by simultaneously increasing RANKL expression and decreasing OPG expression [156]. Furthermore, T cells may be an additional source of RANKL production in bone metastases of multiple myeloma [157]. In addition, a recent study showed that a high-fat diet reduces osteoblast differentiation and OPG expression in prostate cancer patients with bone metastasis and promotes bone metastasis of prostate cancer [158].

Studies have shown that OPG is also expressed in breast cancer cells and tissues, and have identified a negative correlation between OPG and ER expression [159,160]. However, a high serum OPG level is associated with a higher risk of death from breast cancer and is a marker of a poor prognosis for patients with breast cancer [161]. Moreover, studies have shown that overexpressed OPG binds to and inhibits the activity of Apo2L/TRAIL, an apoptosis-inducing ligand of TNF, in vitro, which seems to help breast cancer cells survive, by escaping apoptosis [162]. Furthermore, OPG not only participates in bone metabolism but also promotes the occurrence of the cardiovascular diseases induced by dysregulated vascular mineral metabolism in elderly individuals [163]. Therefore, potential therapeutic strategies designed to inhibit metastatic bone destruction should be developed after considering their negative effects on tumor progression and vascular calcification.

## 8. Therapies for Bone Metastasis

### 8.1. Bone-Modifying Agents

Two classes of drugs, including bisphosphonates (represented by zoledronic acid (ZOL)) and the targeted drug denosumab, are currently approved by the FDA to prevent SREs (bone pain, pathological fractures, hypercalcemia, and bone marrow compression) in patients with bone metastases, delay the time to the first SREs, and reduce subsequent SREs.

#### 8.1.1. ZOL

The use of anti-osteoclastic bisphosphonates to prevent bone loss during adjuvant breast cancer therapy has been validated in multiple clinical trials. Bisphosphonates have been shown to directly inhibit tumor growth and angiogenesis in preclinical models [164]. ZOL is a third-generation bone-targeting bisphosphonate that partially inhibits the absorptive activity of osteoclasts by inhibiting farnesyl diphosphate synthase and protein prenylation [165]. ZOL prevents osteoporosis in postmenopausal women [166], bone loss in breast cancer patients with bone metastases [167], cancer treatment-related bone loss in premenopausal women with early-stage breast cancer [168], and aromatase inhibitor-related musculoskeletal symptoms in postmenopausal women with breast cancer [169]. In terms of adverse events, the CCO-ASCO guidelines state that rare adverse events, such as nephrotoxicity, osteonecrosis of the jaw (ONJ), atypical femoral fractures, and inflammatory eye reactions, have been reported through post-marketing surveillance [170].

In the AZURE (BIG 01/04) trial, the time to first fracture was significantly prolonged in patients with early-stage breast cancer who received standard therapy (neoadjuvant/chemotherapy and/or neoadjuvant/endocrine therapy) for 5 years, with the addition of ZOL every 3–4 weeks for six doses, treatment every 3 months for eight doses, and treatment every 6 months for five doses, and the risk of clinical fracture was reduced [171]. In an open-label, randomized, phase 2 trial, ZOL combined with neoadjuvant chemotherapy for breast cancer reduced the proportion of patients with bone marrow DTCs at the time of surgery, suggesting that ZOL improves the efficacy of neoadjuvant chemotherapy in women with breast cancer, by increasing the clearance of DTCs from bone marrow [172]. A subgroup analysis stratified by menopausal status showed significant differences in the benefit of ZOL. In premenopausal or perimenopausal patients with early breast cancer, disease-free survival (DFS) and OS did not differ significantly between patients treated with ZOL and those treated with standard therapy alone (*n* = 2318). In contrast, adjuvant ZOL treatment improved invasive DFS (IDFS) in patients who were more than 5 years postmenopause at trial entry (*n* = 1041) [173]. After 10 years of follow-up, ZOL still improved the outcomes of postmenopausal women (HR for DFS = 0.82, 95% CI = 0.67–1.00; HR for IDFS = 0.78, 95% CI = 0.64–0.94), and negative MAF expression identified a subgroup of patients who benefited from ZOL treatment, independently of menopausal status [174]. In patients with early-stage breast cancer receiving adjuvant endocrine therapy, the addition of ZOL not only increased the DCR but also prevented bone loss during treatment, and improved bone mineral density at 5 years [175,176].

However, in patients with locally advanced breast cancer, ZOL combined with neoadjuvant chemotherapy had no significant effect on clinical outcomes in the overall cohort and, surprisingly, was also associated with increased extraosseous recurrence and shorter survival in patients with ER+/HER2- cancer who were less than 45 years of age [165]. The benefits of continuous monthly use of ZOL for at least 2 years outweighed the risks in patients with advanced bone tumors, including those related to breast cancer, prostate cancer, and multiple myeloma, according to follow-up studies [177]. In the palliative care setting, continuous second-line zoledronic acid treatment can significantly relieve pain, reduce the n-telopeptide (NTX) level, and exert a relevant palliative care effect on breast cancer patients with bone-related event or progressive bone metastases, after previous first-line bisphosphonate treatment [178].

#### 8.1.2. Denosumab

Denosumab, a human-derived monoclonal antibody targeting RANKL, competes with RANK on the osteoclast surface for RANKL binding, effectively and safely inhibiting bone resorption and reducing the risk of SREs in postmenopausal women with osteoporosis and bone metastasis from multiple myeloma or breast cancer [179,180]. The results of two clinical trials showed no significant difference between denosumab and ZOL in reducing SREs, but denosumab more effectively reduced the levels of UNTX, a marker of urinary bone turnover, to normal levels and was superior to ZOL in suppressing bone turnover [22,181]. Furthermore, a randomized, double-blind study reported that denosumab was superior to zoledronic acid in delaying or preventing SREs in breast cancer patients with bone metastases, and because of its advantages of a mild acute phase response after treatment, no requirement for renal function monitoring, and a convenient administration route of subcutaneous injection, denosumab may be a potential alternative treatment to ZOL for bone metastasis [182,183]. In November 2010, denosumab was approved by the FDA for the treatment of SREs in individuals with bone metastases from solid tumors. One limitation of denosumab is its high cost over the long course of treatment, which may limit its use in the broad population of individuals with bone diseases if its high price is not proportional to its good therapeutic effect [184,185]. In a phase 3 randomized, controlled trial (D-CARE) of neoadjuvant or adjuvant chemotherapy with denosumab or placebo in breast cancer patients, adverse events included osteonecrosis of the jaw (5% vs. <1%), post-treatment hypocalcemia (7% vs. 4%), and a similar incidence of neutropenia [186].

### 8.2. Radiotherapy

#### 8.2.1. External Irradiation

Bone metastasis is the most common indication for palliative RT in breast cancer patients. The therapeutic goals of palliative RT for bone metastases are pain relief, recalcification and stabilization, reduction of spinal cord compression, and minimization of neurological symptoms. For patients with bone metastasis, the ultimate effect of radiation injury is to reduce pain (by interrupting biomolecular pain regulation mechanisms), interrupt osteolytic mechanisms, and reduce tumor burden [187]. Bone modifiers are often used for bone metastasis treatment, and the efficacy of RT combined with bone modifying agents (BMAs) is better than that of BMAs alone [188]. The cumulative incidence of response at 6 months (in which patients with complete or partial responses were considered responders and those with stable or progressive disease were considered non-responders) was 54.4% in the RT + BMA group versus 27.5% in the group treated with BMAs alone. The OS rate in the response group (1 year, 83.1%) was significantly higher than that in the nonresponse group (1 year, 37.5%) (*p* = 0.029). Spinal RT regimens are classified as long-course and short-course. Long-course RT is often administered to patients who are predicted to have a better survival rate, and short-course RT is administered to patients with a poor survival prognosis. Masashi Mizumoto et al. reported a study of 603 patients with spinal metastases who were treated with RT. The median survival times after long- and short-course radiotherapy were 7.9 months and 1.8 months, respectively. The primary tumor site, a good performance status, lack of previous chemotherapy treatment, lack of visceral metastasis, a single bone metastasis, a young age, and a nonhypercalcemic status were associated with a good survival rate [189]. In addition, the triple-negative phenotype was an independent prognostic factor for bone survival (time from first diagnosis of bone metastasis to death) in patients with breast cancer presenting with unstable spinal metastases and who were treated with RT (*p* < 0.001; HR 1.068 [CI 0.933–1.125]) [190]. D. Sit et al. analyzed 376 breast cancer patients who received 464 courses of palliative RT for bone metastasis and found that triple-negative breast cancer was associated with a lower overall response rate (69% vs. 86%, *p* = 0.021) and a lower complete response rate (10% vs. 28.8%, *p* = 0.021). *p* = 0.045) than luminal A breast cancer [191].

Local RT is the standard treatment for metastatic bone pain, and dose fractionation for palliative RT varies worldwide; both single-fraction (SF) and multifraction (MF) regimens are used, and were found to produce similar results for pain control, with complete response rates of 23% and 24%, respectively [192]. Concurrent chemoradiotherapy for painful bone metastases from breast cancer is tolerable and effective. In a prospective study including 84 patients with painful bone metastases from breast cancer, who were randomized to receive a dose of 30 Gy in 10 fractions with or without 825 mg/m^2^ capecitabine every 12 h, the complete response rate to pain at week 4 was 42.9% with capecitabine and 19% without capecitabine, with similar therapeutic toxicity noted in both groups [193].

#### 8.2.2. Radium-223 Dichloride (Radium-223, RA-223)

Radium-223, a bone-like substance with physical properties similar to those of calcium, is the first targeted alpha therapy (TAT) to be approved, in which high-energy alpha particles, not only cause the death of tumor cells by inducing double-stranded DNA breaks, but also disrupt the vicious cycle of bone metastasis, by abolishing the crosstalk between osteoblasts and osteoclasts in the tumor microenvironment [194,195]. Due to the short-range action of alpha particles, TAT exerts a weak toxic effect on adjacent healthy tissues, especially bone marrow, which is useful in the treatment of bone metastases. A report of a phase 3 randomized, double-blind, placebo-controlled trial (ALSYMPCA) indicated that radium-223 was safe and effective in improving OS, with a low bone marrow suppression rate and fewer adverse events in men with castration-resistant prostate cancer (CRPC) and bone metastases [196]. Radium-223 reduces the incidence of symptomatic bone-related events, prolongs the time to occurrence of SREs, significantly reduces the risk of external irradiation for bone pain and bone marrow compression [197], increases the survival rate, significantly improves the quality of life, and slows the decline in the quality of life of patients with CRPC over time [198]. After a 3 year analysis of safety and symptoms, radium-223 remained well tolerated, with no new safety problems emerging [199]. In addition to treating CRPC bone metastasis, radium-223 is also used to treat bone metastasis of advanced breast cancer and advanced renal small cell carcinoma [200,201]. In a mouse model of breast cancer bone metastasis, radium-223 binding to the bone matrix exerted inhibitory effects on the proliferation of breast cancer cells and on osteoblast and osteoclast differentiation, and it prolonged the survival time of mice [202]. A case report showed that radium-223 can be safely applied to breast cancer patients with hormone-refractory bone metastases. Bone pain was relieved after treatment, and tumor markers were reduced accordingly, providing a clinical basis for further research [203]. The results of a phase 2 trial (NCT02366130) showed that radium-223 combined with hormone therapy may be effective in treating bone metastases from HR+ breast cancer, with tolerable adverse events [204].

### 8.3. Surgery

Most surgeries provide palliative treatment to relieve pain, stabilize bone metastases, restore weight-bearing ability and functional activity, and improve quality of life. A small number of isolated bone metastases may be cured by extensive resection. The choice of the surgical method and extent of resection must be agreed upon by clinicians from multiple disciplines, such as oncology, orthopedics, and radiotherapy, based on the patient’s prognosis, tumor location, and general condition [205]. Since the prognosis of some solid tumors (such as breast cancer, prostate cancer, and thyroid cancer) is good [206], wide resection of solitary bone metastases is considered to be a reasonable approach and may improve the overall prognosis of patients [207]. For patients with multiple metastases, particularly visceral and brain metastases, the survival rate is reduced, and the choice of surgical intervention should be determined cautiously [208]. Emergency surgical indications for bone metastases include neurological deficits or paraplegia caused by spinal metastases and pathological fractures, and surgical indications for fracture risk and refractory pain are evaluated as selective. Notably, the patient’s life expectancy and prognosis should be considered when evaluating the choice of surgery [209].

### 8.4. Immunotherapy

Novel targeted ICI therapies, including anti-PD-1 and anti-PD-L1 monoclonal antibodies, have shown efficacy against tumors such as lung cancer, head and neck squamous cell carcinoma, triple-negative breast cancer, and malignant melanoma [210,211,212,213]. An animal study indicated that anti-PD-1 therapy significantly inhibited the growth of triple-negative breast cancer cells and prolonged the survival of mice [214]. Experiments using mice bearing Lewis lung cancer bone metastases showed that anti-PD-1 therapy provides long-term benefits in preventing bone destruction and reducing bone cancer pain, by inhibiting osteoclast production, although baseline pain sensitivity is transiently increased [215]. The utility of anti-PD-1 as a treatment for TNBC has also been documented in clinical trials. The Impassion-130 trial (NCT02425891), in which patients were randomized to receive albumin-bound paclitaxel or albumin-bound paclitaxel plus atezolizumab as a first-line treatment for metastatic triple-negative breast cancer, showed a significant median progression-free survival (PFS) benefit in the immunotherapy group compared to the control group (7.2 months vs. 5.5 months). The median PFS for patients with PD-L1-positive tumors was 7.5 months in the atezolizumab group and 5.0 months in the placebo group [216]. In contrast, in the Impassion 131 trial, paclitaxel plus atezolizumab did not produce a significant difference in PFS among PD-L1-positive or intention-to-treat groups of patients with advanced triple-negative breast cancer who had completed adjuvant chemotherapy or had not received any therapy [217]. In the KEYNOTE-355 trial, pembrolizumab combined with chemotherapy improved PFS compared with the placebo in patients with PD-L1-positive metastatic triple negative breast cancer (9.7 months vs. 5.6 months; HR, 0.65; 95% CI, 0.49 to 0.86; *p* = 0.0012), but no improvement in PFS was observed in patients with lower PD-L1 expression [218]. The combination of atezolizumab and chemotherapy was generally well tolerated, and thyroid dysfunction and rash were the most common immune-related adverse events [219]. Unfortunately, immunotherapy specifically designed to inhibit bone metastases or bone lesion processes has been less extensively studied. However, ICIs may have a role in inhibiting bone metastases, in view of prolonging the PFS of patients with PD-L1-positive metastatic triple-negative breast cancer.

### 8.5. Other Factors Related to Bone Transformation

#### 8.5.1. PTHrP Inhibitors

PTHrP is expressed at high levels in breast cancer cells and plays an important role in activating osteoblasts and stromal cells to express RANKL, which is an important link mediating osteolytic bone destruction. Saito H et al. found that an anti-PTHrP monoclonal antibody effectively inhibited the osteolytic metastasis of breast cancer cells [220]. Human antibodies against PTHrP have also been shown to be beneficial in the treatment of cancer-related hypercalcemia and cachexia [221]. Clinical trials of the human anti-PTHrP antibody CAL compared with ZOL for the treatment of breast cancer bone metastasis have been conducted, but no reports are available on the safety and efficacy of CAL. Thus, human anti-PTHrP antibodies seem to be a hotspot for future research on bone metastasis.

#### 8.5.2. TGF-β Blockers

TGF-β is released from the bone matrix through the action of osteoclasts and promotes metastatic growth, which is a key factor contributing to the vicious cycle of bone destruction. Therefore, TGF-β blockers are theoretically among the potential agents of choice for the treatment of breast cancer bone metastasis. Galunisertib (LY2157299), a small molecule inhibitor of the TGF-β R1 serine/threonine kinase, was shown to have acceptable tolerability and safety in a phase 1 study of Japanese patients with advanced solid tumors [222]. In another clinical study, blockade of TGFβ-R1 with galunisertib enhanced the efficacy of the anti-GD2 antibody dinutuximab (CH14.18) and activated NK cells to treat neuroblastoma [223]. In addition, galunisertib exerts potential therapeutic effects on rectal cancer, prostate cancer, and gynecological tumors [224,225,226]. Currently, no large-scale clinical studies have reported the application of TGF-β blockers in patients with bone metastases. Thus, the therapeutic value and population that may benefit from TGF-β blockers deserve further study.

## 9. Conclusions and Perspectives

This review is a novel topic in recent years, combining the scientific and clinical fields of bone metastasis of breast cancer, providing research directions for the molecular mechanism of bone metastasis of breast cancer, and laying a theoretical foundation for accurate treatment. The elaboration and discussion provided above further expand the “seed and soil” hypothesis of bone metastasis, which states that the process of bone metastasis is the result of mutual selection and interactions between tumor cells and the bone microenvironment, beginning even before tumor cells reach the bone marrow. The characteristics of distant invasion and metastasis are related to gene expression in primary tumor cells. The identification of serum markers associated with a high metastasis risk will be helpful for guiding the selection of treatment, in terms of time and population. After reaching the bone marrow, breast cancer cells may remain dormant for several years before metastasis becomes detectable, to protect themselves from the tumoricidal effect of the immune microenvironment. Drugs that target tumor cells in the bone microenvironment, to enhance antitumor efficacy, must be developed and applied. The vicious cycle of osteolytic bone destruction induced by tumor cells is accompanied by intercellular interactions and the regulation of the immune microenvironment, which is interconnected through various complex signaling pathways. Therefore, the identification of key targets to block this vicious cycle is both a challenge and direction for the treatment of breast cancer bone metastasis. In addition, targeted immunotherapy, targeted alpha therapy, and targeted genetic engineering are worthy of study.

## Data Availability

Not applicable.
